# Metal element fingerprints combined with chemometrics deciphering the discrimination of different Calculus bovis and a novel risk–benefit assessment

**DOI:** 10.3389/fchem.2026.1752261

**Published:** 2026-02-10

**Authors:** Tian-Tian Zuo, Zhao Wang, Yuan-Sheng Guo, Hong-Yu Jin, Li-Na Liu, Jing Liu, Xianlong Cheng, Feng Wei, Yong-Qiang Lin

**Affiliations:** National Institutes for Food and Drug Control, State Key Laboratory of Drug Regulatory Science, Beijing, China

**Keywords:** Calculus bovis, chemometrics, multielement fingerprinting, risk–benefit assessment, species identification

## Abstract

In recent years, the phenomenon of adulterated Calculus bovis being sold as cheap Calculus bovis has been continuously observed in the market. To effectively differentiate among the three products, original Calculus bovis, Bovis calculus sativus, and Bovis calculus artifactus, our group analyzed and evaluated the elements present in these products that are relevant to the therapeutic efficacy of traditional Chinese medicines (TCMs). The elements were evaluated using inductively coupled plasma mass spectrometry (ICP-MS) combined with chemometric analysis to differentiate among the abovementioned three marketed products. Chemometric analysis showed that Calculus bovis samples could be well separated based on established multi-element fingerprints, with Sr, Ca, K, V, Ti, and Al identified as the most important variables for discrimination. When Calculus bovis is medicinally used or consumed, non-carcinogenic risk assessment results indicate that the risks associated with exposure to heavy metals such as Pb, Cd, As, and Hg were acceptable for both genders. However, the hazard index (HI) of Pb in Calculus bovis exceeded 1. The results of the carcinogenicity risk assessment indicated that the carcinogenicity risk (CR) of Pb and As in all Calculus bovis samples was less than 1, indicating that the risks were acceptable. For the nutrients K, Ca, and Mg, the combined risk–benefit evaluation of the three Calculus bovis products indicated that the HI exceeded 1 across all three products, regardless of the gender of the individual consuming the medication. The benefit index (BI) for K, Mg, Cr, Se, and Mo was less than 1, indicating that risks need further consideration. The identification model developed in this study can effectively differentiate between the three marketed Calculus bovis products, aiming to address quality issues such as adulteration, forgery, under-injection, and other such concerns.

## 1 Introduction

Calculus bovis, which is the dried gallstone obtained from bovine animals' gallbladder or bile duct, is extremely valuable in traditional Chinese medicine (TCM) therapies. Calculus bovis, one of the traditional Chinese medicinal materials (CHMs), has been clinically used for more than two thousand years (Wu et al., 2016; Yu et al., 2018; Zhong et al., 2016). It is renowned for its anticonvulsant and antiepileptic properties, along with sedation, hepatoprotection, heat-clearing, detoxification, and other effects. Because Calculus bovis is expensive and rare, with a high demand in the market, substitutes for Calculus bovis have been developed, including Bovis calculus sativus and Bovis calculus artifactus (Li et al., 2018; Liu et al., 2020; Yu et al., 2020). Calculus bovis, Bovis calculus sativus, and Bovis calculus artifactus are all mentioned in the 2020 edition of the Chinese Pharmacopoeia. The sources of these three products are different. Calculus bovis is derived from the dried gallstones of bovine animals. Bovis calculus sativus is prepared from the bile of the bovine family and contains deoxycholic acid, cholic acid, calcium bilirubin complex, and other components. Bovis calculus artifactus contains bovine bile powder, cholic acid, porcine deoxycholic acid, taurine, bilirubin, cholesterol, trace elements, and other synthetic compounds. The 2020 version of the Chinese Pharmacopoeia lists different compositions of the three Calculus bovis products. The bilirubin (C_33_H_36_N_4_O_6_) content of Calculus bovis and Bovis calculus sativus should not be less than 25.0% and 35.0%, respectively. In contrast, the bilirubin content of Bovis calculus artifactus should not be less than 0.63%, much lower than that in the other two types of Calculus bovis; this requires extra attention (Kong et al., 2012; Qiao et al., 2011). In addition, taurine, which has attracted considerable attention in recent years, was originally discovered in bovine bile and is now added to many energy drinks and sports supplements because of its performance-enhancing properties. Due to the different sources and different contents of the characteristic components of the three Calculus bovis products, the prices vary greatly (300,000–400,000 RMB/kg for Calculus bovis, 40,000–50,000 RMB/kg for Bovis calculus sativus, and several hundred RMB/kg for Bovis calculus artifactus). The use of cheap Calculus bovis substitutes to replace or adulterate Calculus bovis has emerged in the market recently, leading to problems such as insufficient dosing of Chinese patent medicines containing Calculus bovis (including Calculus bovis antidote tablets and Calculus bovis Qianjin San) (Liu et al., 2015; Phansalkar et al., 2017). These problems have seriously affected the quality of Calculus bovis formulations and their Calculus bovis-containing proprietary Chinese medicines, jeopardizing the consumers’ benefits. Identifying and differentiating the three forms of Calculus bovis hold much significance.

Due to significant price differences among Calculus bovis products, various product identification methods have been developed. In previous studies, identification relied mainly on morphological, microscopic, physicochemical, and empirical methods, proving effective over time. However, the methods are often time-consuming and labor-intensive, requiring personnel with specific skills, thus affecting the long-term development of TCM and food sectors to some extent. New technologies have been explored recently to distinguish different Calculus bovis products, including UHPLC-Q/TOF-MS combined with principal component analysis (PCA), showing potential in distinguishing three Calculus bovis products. However, further research is needed on characteristic biomarkers for accurate classification (Liu et al., 2020). Some researchers used near-infrared spectroscopy to differentiate between different Calculus bovis products, offering new insights into Calculus bovis identification (Tian et al., 2024). HPLC and UPLC-MS have also been used for Calculus bovis identification (Liu et al., 2015; Shimada et al., 2013). Although these methods provide various approaches for Calculus bovis identification, the chemical composition of Calculus bovis is influenced by external factors and may not fully meet market demands. Therefore, we need to develop identification methods that are highly accurate, fast, and easy to operate, to achieve precise differentiation of different Calculus bovis products.

The therapeutic effects of TCMs and food supplements are related to their organic components and closely related to their elements (Janisse et al., 2023). Most metallic elements exist stably in natural products. Nutrient elements are closely related to the quality and therapeutic efficacy of herbal medicines. The inorganic profile of TCMs can be considered more stable than the organic profile (due to changes induced by metabolism or industrial processing on the organic compounds present). On the other hand, herbs may be contaminated by cadmium (Cd), lead (Pb), arsenic (As), mercury (Hg), and other metal elements during planting, harvesting, and processing, which pose serious risks to human health (Aaseth, 2022; Himoto and Masaki, 2020). The profile of chemical elements may add essential information on the TCMs' type and can be used as a prime tool to distinguish the authenticity and quality of TCMs.

Considering the popularity of Calculus bovis used in TCMs and food supplements in China and other countries around the world as food and medicine and given the increasing concerns on the authenticity and safety of Calculus bovis, we aimed (1) to determine the contents of 28 heavy metals and trace elements in the three Calculus bovis products using inductively coupled plasma mass spectrometry (ICP-MS) to determine the content and distribution pattern of the contents of the inorganic element; (2) to differentiate between three Calculus bovis products, using chemometric analysis, including cluster analysis, principal component analysis (PCA), partial least squares (PLS), and self-organized mapping artificial neural network (SOM); (3) to establish a novel multi-element risk–benefit assessment strategy to comprehensively evaluate the risks and benefits of Calculus bovis types.

## 2 Materials and methods

### 2.1 Sample collection

This study examined five batches of Calculus bovis, fifteen batches of Bovis calculus sativus, and five batches of Bovis calculus artifactus. Dr. Shuai Kang authenticated all the samples. Furthermore, 10 batches of samples were used as the training set, comprising three batches of Calculus bovis (numbered V1–V3), four batches of Bovis calculus sativus (numbered V4–V6), and three batches of Bovis calculus artifactus (numbered V7–V10), which were used as the test set to verify the validity of the established model.

### 2.2 Sample preparation for determining the metal content

The sample was crushed and passed through a 50-mesh sieve. The sample powder (0.25 g) was mixed with 5.0 mL HNO_3_and placed in a microwave system to be digested for 3 minutes to 120 °C, 2 minutes from 120 °C to 150 °C and held for 3 minutes, and 2 minutes from 150 °C to 190 °C and held for 12 min. The ablated sample was then transferred to a 25-mL measuring flask; the volume was made up with ultrapure water and then measured. A blank solution was prepared at the same time.

### 2.3 ICP-MS analysis

A total of 28 metals, namely, Al, As, B, Ba, Be, Ca, Co, Cu, Cr, Cd, Fe, Hg, K, Mg, Mn, Mo, Na, Ni, Pb, S, Sb, Se, Sr, Sn, Ti, Tl, V, and Zn were analyzed using an Agilent 7700X ICP-MS (Agilent 7700X, Agilent Technologies Co., USA). After appropriate dilution, the instrument was tuned and calibrated using tuning solutions (containing Li, Ge, Rh, In, Tb, Lu, and Bi) and ICP multi-element standard solutions containing the above-mentioned elements.

The optimum instrumental conditions for ICP-MS measurement are as follows: plasma gas flow rate: 15.0 L/min*;* peristaltic pump 0.20 r/s; nebulization chamber temperature: 2 °C; auxiliary gas flow rate: 0.8 L/min; He gas flow rate: 5 mL/min; carrier gas flow rate: 0.8 L/min; RF power: 1550 W; data sampling mode: peak hopping acquisition mode; sampling depth: 10 mm; repetition: 3 times; scanning: 100 times.

### 2.4 Calibration procedure

Elements in Calculus bovis samples were quantitatively analyzed using calibration curves obtained by diluting a stock standard solution of elements. Under optimized measurement conditions, six different concentrations (0.1, 1, 2, 5, 10, and 20 μg/L) of Be, Sb, Mo, S, Sn, and Hg; different concentrations (1, 10, 20, 50, 100, and 200 μg/L) of As, Cd, Co, Cr, Ni, Pb, Se, TI, and V; different concentrations (10, 100, 200, 500, 1,000, and 2000 μg/L) of B, Ba, Cu, Mn, Ti, and Zn; different concentrations (20, 200, 400, 1,000, 2,000, and 4,000 μg/L) of Sr; different concentrations (100, 1,000, 2000, 5,000, 10,000, and 20,000 μg/L) of Al and Fe; and different concentrations (400, 4,000, 8,000, 20,000, 40,000, and 80,000 μg/L) of Ca, K, Mg, and Na were measured, and calibration curves were plotted.

### 2.5 LOD and LOQ

The blank sample was used for estimating LOD and LOQ. The LODs were calculated as triple standard deviation (σ) of the signal obtained from a set of independently prepared reagent blanks (n = 10), and the LOQs were 10σ. The values of LOD and LOQ are shown in Supplementary Table S1.

### 2.6 Precision

The RSD of each element was calculated from the peak area value of each element by taking the solution at point 4 of the standard curve and injecting the sample six times consecutively; the RSD of each element was in the range of 2.4%–3.6%, indicating that the precision of the instrument was good.

### 2.7 Spiked recovery

To approximately 0.25 g of powdered Bovis calculus sativus,1 mL of the mixed standard solution was added at the following mass concentrations: Be, Sb, Mo, S, Sn, and Hg at 2 μg/L; As, Cd, Co, Cr, Ni, Pb, Se, TI, and V at 20 μg/L; B, Ba, Cu, Mn, Ti, and Zn at 200 μg/L; Sr at 400 μg/L; Al and Fe at 2,000 μg/L; and Ca, K, Mg, and Na at 8,000 μg/L. All samples were tested according to the conditions described in *Section 2.3*, and the recoveries were calculated, showing that the recoveries of the 28 elements were 72.6–117.4%, meeting the requirements for trace detection.

### 2.8 Establishment of a risk–benefit assessment strategy for three species of Calculus bovis

Previous research categorized elements as hazardous and nutrients, but the studies generally focused only on the health risks of heavy metals and hazardous elements, with less attention given to the health risks posed by nutrients to the human body. Nutrients provide health benefits to the human body and, hence, need attention (Chowdhury et al., 2024; Ma et al., 2022; Nag and Cummins, 2022). To summarize, the health risk assessment should be carried out for heavy metals and harmful elements, and for nutrients, health risks and benefits should be considered. Risk–benefit evaluation should consider both assessments. In addition, a health risk assessment system and a risk–benefit evaluation system consistent with the characteristics of TCM use should be established in the assessment process to ensure the scientific basis and rationality of the risk assessment to the greatest extent possible.

#### 2.8.1 Non-carcinogenic risk–benefit assessment

According to the principles of food safety risk assessment guidance, the risk assessment model proposed here follows the basic procedures of risk assessment, including hazard identification, hazard characterization, exposure assessment, and risk characterization (Zuo et al., 2020; Zuo et al., 2023). This study comprehensively assessed the risks from both medicinal and dietary perspectives. Hazard characterization determines dose–response relationships or health-based guidance values (HBGVs) based on potential adverse effects produced by each element. The established HBGVs for elements Pb, Cd, As, Hg, Cu, Al, Mn, and Ni are 3.5, 1.0, 0.3, 0.6, 500.0, 1,000.0, 140.0, and 20.0 μg/(kg·d), respectively. Elements without HBGV are referenced from the “Chinese Dietary Reference Intakes (2023 Edition)” setting provisional tolerable daily intakes (PTDIs) for K, Ca, Na, Mg, Fe, Zn, Cr, Se, Mo, V, Ba, and Be at 31.6, 28.7, 23.0, 4.7, 0.6, 0.6, 0.4, 5.7, 12.9, 9.0, 200.0, and 2.0 μg/(kg·d), respectively. To calculate the non-carcinogenic risk, the following equation was applied:

$$\mathrm{Exp}=\frac{EF\times Ed\times IR\times C}{W\times AT}.$$ (1)

Considering the dual medicinal and dietary use of Calculus bovis, exposure levels for elements are calculated under two scenarios: (1) medicinal use: in Formula 1, Exp represents the daily exposure of elements per kilogram of body weight (μg/kg). *EF* denotes the exposure frequency of medicinal herbs, with P_95_ set at 90 days/year based on effective consumer surveys across 11 provinces. *Ed* reflects the exposure duration over an herbal medicine lifespan that typically does not exceed 20 years. *IR* indicates the daily intake of medicinal herbs (g/d), following dosage guidelines from the 2020 ″Chinese Pharmacopoeia” edition. *C* represents the residual amount of metallic elements in herbs (mg/kg). *W* represents the human body weight; according to statistics from the National Health Commission, the average weight in China in 2020 was 69.6 kg for male and 59 kg for female individuals. *AT* denotes the average lifespan in days, calculated at 70 years (25,550 days). (2) Edible use: According to survey data, *EF* and exposure duration are 260 days/year and an average lifespan of 70 years (*Ed*), respectively. *IR* reflects the daily intake of seaweed as a food source, with research indicating that it is commonly consumed as a vegetable in most regions, setting the IR at 0.5 g/d. Other parameters are the same as in the medicinal scenario.

Risk–benefit characterization involves both risk and benefit characterization. This entails a comprehensive analysis of assessment results, balancing risks and benefits and providing scientific recommendations to risk managers. The level of risk posed by elements involved in the risk assessment is described using a hazard index (HI), calculated according to the following formula:

$$HI=\mathrm{Exp}\times \frac{SF}{PTDI}.$$ (2)

Using Equation 2 and the risk characteristics of Pb, Cd, As, Hg, Cu, K, Ca, Na, Mg, Fe, Zn, Cr, Se, Mo, V, Ba, and Be in Calculus bovis, we calculated the elements’ HI for risk assessment. When used as medicine, the safety factor (SF) is 10; when used as food, the SF is 1; PTDI is the provisional tolerable daily intake of the element. The PTDI values for Pb, Cd, As, Cu, Al, Mn, and Ni are consistent with their HBGVs. If HI ≤ 1, the health risk is low, and the risk is acceptable; if HI > 1, the risk is high and should be taken seriously.

For risk–benefit assessment, the benefits of the nutrient elements, including K, Ca, Na, Mg, Fe, Cu, Zn, Cr, Se, and Mo, need to be considered. The BIs were calculated using Equation3.

BI=Exp×SF/RDI (3)

In Equation3, Exp is the exposure calculated using Equation 1, HI and SF have the same definition as in Equation 2, and PTDI is the provisional tolerable daily dose of the element. For male individuals, the PTDI values for K, Ca, Na, Mg, Fe, Cu, Zn, Cr, Se, and Mo are 31.6, 28.7, 23.0, 4.7, 0.6, 0.1, 0.6, 0.4, 5.7, and 12.9 mg/kg/day, respectively. For female individuals, the PTDI values for K, Ca, Na, Mg, Fe, Cu, Zn, Cr, Se, and Mo are 37.3, 33.9, 27.1, 5.6, 0.7, 0.1, 0.7, 0.5, 6.8, and 15.3 mg/kg/day, respectively. RDI is the recommended daily intake dose, determined according to the Reference Intake of Dietary Nutrients for Chinese Residents issued by the Health Industry Standard of the People’s Republic of China. For male individuals, the RDI values for K, Ca, Na, Mg, Fe, Cu, Zn, Cr, Se, and Mo are 31.6, 14.4, 23.0, 4.0, 0.2, 0.0, 0.1, 0.4, 0.7, and 1.2 mg/kg/day, respectively; for female individuals, the RDI values for K, Ca, Na, Mg, Fe, Cu, Zn, Cr, Se, and Mo are 37.3, 16.9, 27.1, 4.7, 0.3, 0.0, 0.2, 0.5, 0.8, 1.4 mg/kg/day, respectively. If BI < 1, the health benefit is low; if BI ≤ 1 ≤ HI, the risk and benefit are balanced. If HI > 1, the health risk is unacceptable.

#### 2.8.2 Carcinogenic risk assessment

For carcinogenic risk assessment, the lifetime cancer risk (CR) for carcinogenic heavy metals was calculated using the cancer slope factor (CSF). The lifetime CR is described as the probability of a person developing cancer throughout the lifetime as a result of exposure to specific heavy metals. The following equation defines CR:

CR=CDI×CSF×0.001. (4)

*CSF* is the oral cancer slope factor. According to the Integrated Risk Information System database, the *CSF*s for As and Pb were assumed to be 1.5 and 8.5 × 10^−3^ mg/kg/day, respectively. Other parameters in Equation 3 are shown in Equations 1, 2 [Disp-formula e2]. The acceptable *CR* standard ranges from 10^–6^ to 10^–4^. Therefore, if the *CR* value is >10^–4^, the carcinogenic risk over a lifetime is considered unacceptable.

### 2.9 Data processing and chemometric analysis

PCA, partial least squares discriminant analysis (PLS-DA), and other chemometric analyses were conducted using ChemPattern 2017 Pro software (Chemmind, China). Figures were plotted using GraphPad 5.0 (San Diego, CA, USA). Statistical analyses, including means, standard deviations, and ranges, were calculated in Microsoft Excel 2019 (Microsoft Co, USA).

## 3 Results

### 3.1 Multi-element analysis

#### 3.1.1 Establishment of elemental fingerprints of Calculus bovis

This study analyzed 28 inorganic elements found in Calculus bovis. A fingerprint map of inorganic elements in Calculus bovis was established. Table 1 presents the average content of these 28 inorganic elements across three Calculus bovis varieties. Among all elements, the 28 metallic elements were categorized into four groups: macronutrients, essential trace elements, trace elements (non-essential), and hazardous elements.

**TABLE 1:** The mean values (mg/kg) of the 28 elements in Calculus bovis from different species.

Element	Bovis calculus artifactus (n = 5)		Bovis calculus (n = 5)		Bovis calculus sativus (n = 15)	
	Mean	Range	Mean	Range	Mean	Range
K	869.3	695.2–1,124.4	676.0	548.8–840.4	1,742.8	1,613.5–1,852.6
Ca	14,818.0	10,590.6–18,831.7	14,650.0	13,002.4–16,889.3	55,399.6	53,110.3–60,275.2
Na	12,755.0	9,176.1–14,613.7	9,582.6	8,637.4–11,292.0	9,797.9	8,754.9–10,657.9
Mg	256.8	94.8–515.2	1,084.4	186.7–2,427.3	535.9	505.7–574.0
Fe	93.565	46.517–151.874	1,111.095	85.773–4,207.325	180.179	164.674–202.429
Cu	2.069	1.679–2.728	67.958	28.869–108.165	6.242	5.883–7.015
Zn	1,549.07	57.859–2,863.097	1,160.66	866.899–1,315.466	393.50	358.023–568.552
Mn	3.660	3.089–4.677	438.464	257.728–743.304	17.165	16.147–18.753
Cr	2.176	1.013–4.352	8.858	5.782–14.380	3.504	2.934–4.350
Se	0.244	0.219–0.269	1.263	1.073–1.517	0.934	0.825–1.026
Co	0.115	0.094–0.143	0.864	0.187–2.876	0.443	0.361–0.637
Mo	0.114	0.077–0.164	0.959	0.550–1.286	0.171	0.147–0.199
V	0.082	0.051–0.120	0.186	0.118–0.298	0.553	0.511–0.589
Ni	1.640	0.994–2.410	4.698	2.457–6.056	1.723	1.364–2.130
Sr	8.021	3.620–12.981	4.454	2.155–8.160	106.350	100.261–111.276
S	40.081	34.494–43.296	13.096	10.937–15.113	20.863	19.111–23.159
Sn	0.178	0.154–0.206	0.332	0.140–0.871	0.312	0.295–0.331
B	2.614	1.819–3.720	3.914	1.768–6.143	2.414	1.807–4.820
Ba	1.293	1.170–1.462	10.355	1.466–37.624	1.813	1.541–2.159
Ti	1.362	0.643–2.190	1.391	0.954–1.939	6.791	5.870–8.543
Be	0.007	0.000–0.018	0.065	0.003–0.275	0.022	0.012–0.044
Al	17.517	6.959–29.016	35.965	11.215–99.722	102.621	97.728–109.346
Sb	0.013	0.012–0.015	0.115	0.021–0.435	0.025	0.023–0.028
Tl	0.006	0.003–0.017	0.193	0.123–0.264	0.001	0.001–0.002
As	0.582	0.184–0.890	1.983	0.748–3.824	0.969	0.662–2.257
Cd	0.021	0.004–0.077	0.092	0.068–0.140	0.046	0.042–0.051
Hg	0.205	0.182–0.227	0.897	0.191–2.181	0.084	0.075–0.098
Pb	0.403	0.124–0.927	219.262	4.746–1,023.194	0.291	0.240–0.360

#### 3.1.2 Study of elemental contents in Calculus bovis from different species

**FIGURE 1:**
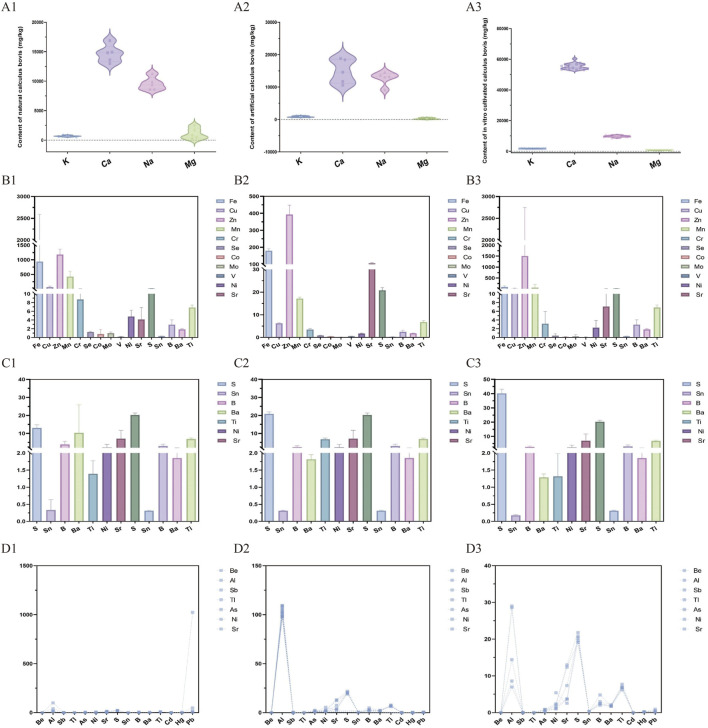
The contents (mg/kg) of macronutrients **(A1-A3)**, essential trace elements **(B1-B3)**, trace elements (non-essential, **(C1-C3)**), and hazardous elements **(D1-D3)** in the Calculus bovis, Bovis calculus sativus, and Bovis calculus artifactus samples.

The first group consisted of K, Ca, Na, and Mg (Figures 1A1–A3). In general, the contents of these elements in the three species of Calculus bovis followed the pattern Ca > Na > K > Mg. Among these, K regulates osmotic pressure and maintains the acid–base balance of the body (Balčiauskas et al., 2022). Ca promotes bone growth and development, maintaining the normal blood supply function of the heart and nerve and muscle activities (Ma et al., 2019; Torti and Torti, 2020). Na maintains the acid–base balance of the body, and Mg regulates the vitality of nerves and muscles (Bobulescu and Moe, 2006; Gagnon and Delpire, 2020; Gröber et al., 2015). The K and Ca contents were highest in Bovis calculus sativus (1,742.8 and 55,399.6 mg/kg, respectively), followed by Bovis calculus artifactus and Calculus bovis. The Na content was highest in Bovis calculus artifactus (12,755.0 mg/kg), followed by Bovis calculus sativus and Calculus bovis. By contrast, the Mg content was highest in Calculus bovis (1,084.4 mg/kg), followed by Bovis calculus sativus and Bovis calculus artifactus.

The second group of essential trace elements consisted of Fe, Cu, Zn, Mn, Cr, Se, Co, Mo, V, Ni, and Sr, with the average contents ranging from 778.04 to 0.32 mg/kg. In general, the contents of these elements in the three species of Calculus bovis followed the order of Zn > Fe > Mn > Sr > Cu > Cr > Ni > Se >Co> V > Mo (Figures 1B1–B3). The average contents of Fe, Cu, Mn, Cr, Se, Co, Mo, and Ni were highest in Calculus bovis (1,111.095, 67.958, 438.464, 8.858, 1.263, 0.864, 0.959, and 4.698 mg/kg, respectively). The average content of Zn was highest in Bovis calculus artifactus (1,549.07 mg/kg). In contrast, the average contents of V and Sr were highest in Bovis calculus sativus (0.553 and 106.350 mg/kg, respectively).

The third group consisted of non-essential trace elements, including S, Sn, B, Ba, and Ti, with the average contents ranging from 0.29 to 23.15 mg/kg. The average contents of elements in this group followed the order of S > Ti > Ba > B > Sn (Figures 1C1–C3). The average content of S was highest in Bovis calculus artifactus (40.081 mg/kg). The average contents of Sn, B, and Ba were highest in Calculus bovis (0.332, 3.914, and 10.355 mg/kg, respectively). In contrast, the average content of Ti was highest in Bovis calculus sativus (6.791 mg/kg).

The last group comprised eight harmful elements: Be, Al, Sb, Tl, As, Cd, Hg, and Pb. The average contents of Al, Pb, and As were 72.269, 44.108, and 1.094 mg/kg, respectively, while the contents of the remaining elements were below 1 mg/kg (Figures 1D1–D3). The average contents of these elements, except for Al, were highest in Calculus bovis (0.065, 0.115, 0.193, 1.983, 0.092, 0.897, and 219.262 mg/kg for Be, Sb, Tl, As, Cd, Hg, and Pb, respectively). The average content of Al was highest in Bovis calculus sativus (102.621 mg/kg). The average contents of Be, Al, Sb, As, and Cd were lowest in Bovis calculus artifactus (0.007, 17.517, 0.013, 0.582, and 0.021 mg/kg, respectively). The average contents of Ti, Hg, and Pb were lowest in Bovis calculus sativus (0.001, 0.084, and 0.291 mg/kg, respectively).

The 2020 edition of the Chinese Pharmacopoeia of the People’s Republic of China sets the following limits for inclusion of heavy metals in plant medicines: Pb ≤ 5 mg/kg, Cd ≤ 1 mg/kg, As ≤ 2 mg/kg, Hg ≤ 0.2 mg/kg, and Cu ≤ 20 mg/kg. According to the results of this study (Table 1), the contents of Pb and Hg in four batches of Calculus bovis exceeded the limits, the contents of As in two batches exceeded the limits, and the contents of Cu in five batches all exceeded the limits. The As content in one batch of Bovis calculus sativus and the contents of Hg in two batches of Bovis calculus artifactus were slightly higher than the limits. Thus, in this study, the risk–benefit assessment of Calculus bovis is of great significance.

#### 3.1.3 Chemometric analysis of elemental fingerprints

The general features of the 28 metals revealed that different types of Calculus bovis varied in metal contents. We preliminarily concluded that Calculus bovis samples have specific metal element profiling (SMEP), which could be used to identify and describe their characteristics. Herein, chemometric methods were applied to simplify the experimental information and obtain clearer visual results.

The data on inorganic element contents from three species of Calculus bovis were imported into ChemPattern 2017 Pro software in a standardized format. The measured values of elements were subjected to normalization preprocessing, followed by multivariate statistical analysis and modeling such as cluster heat map analysis, PCA, PLS-DA, and SOM.

##### 3.1.3.1 Hierarchical cluster analysis (HCA)

**FIGURE 2:**
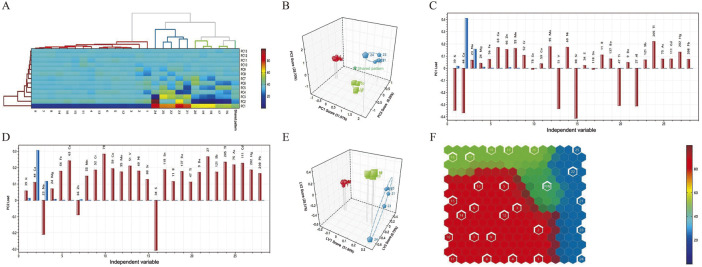
Dendrograms of the HCA **(A)**; score plots of the PCA model **(B)**; loading plots of the elements in PC1 **(C)**; loading plots of the elements in PC2 **(D)**; score plots of the PLS-DA model **(E)**; self-organizing mapping artificial neural network analysis **(F)**.

Hierarchical cluster analysis (HCA) is a common unsupervised learning method used to group objects in a dataset into multiple clusters based on certain similarity criteria. Objects within the same cluster are similar to each other in some sense, while there are significant differences between objects in different clusters. Based on data differences between samples, Calculus bovis with similar properties were systematically clustered. The systematic cluster diagrams and two-dimensional cluster diagrams of the three samples are shown in Figure 2A. The samples were clustered horizontally, while the contents of inorganic elements were clustered vertically. The difference in the color of the heat map was used to reflect the magnitude of the residual amounts of the corresponding inorganic elements in Calculus bovis. Cluster analysis of the 28 inorganic element residues showed that the three Calculus bovis samples were clustered into three categories, consistent with the Calculus bovis categories.

##### 3.1.3.2 Principal component analysis

PCA is an unsupervised dimensionality reduction technique that reduces complex multidimensional data to two or three principal components, which can then be visualized through scatter plots. As shown in Figure 2B, the three Calculus bovis samples were generally distributed in different spatial locations, and the different types of Calculus bovis could be well distinguished. For the PCA, the cumulative variance contribution rates of the first three principal components, PC1, PC2, and PC3, were 88.95%. In addition, based on loadings (Figures 2C,D), it was possible to determine the indicators that led to the differences between the different Calculus bovis samples (i.e., the characteristic inorganic elements). The characteristic inorganic elements in the PC1 direction were Sr, Ca, K, V, Ti, and Al, while the characteristic inorganic elements in the PC2 direction were S, Se, Al, Cu, and Na.

##### 3.1.3.3 Partial least squares-discriminant analysis

PLS-DA is a supervised classification method that constructs a classification model for different Calculus bovis samples by extracting the maximum variance and minimizing the errors between categories. We chose PLS-DA to analyze data on the content of inorganic elements in the three species of Calculus bovis; the model score plots are shown in Figure 2E. In the plots, the samples of Calculus bovis, Bovis calculus sativus, and Bovis calculus artifactus were clustered into one category, indicating that the different categories of samples were better distinguished.

##### 3.1.3.4 Self-organizing mapping artificial neural network analysis

Artificial neural networks have been a research hotspot in artificial intelligence since the 1980s. A self-organized artificial neural network is a nonlinear dimensionality reduction method that reduces the dimensionality of high-dimensional data by simulating the neuronal process in the human brain corresponding to external stimuli, the principle of which belongs to the nonlinear PCA analysis. The mapping of different species of Calculus bovis in the space of neurons agrees with the results of cluster analysis and PCA, and the result is more visually intuitive (Figure 2F).

#### 3.1.4 Experimental model validation

**FIGURE 3:**
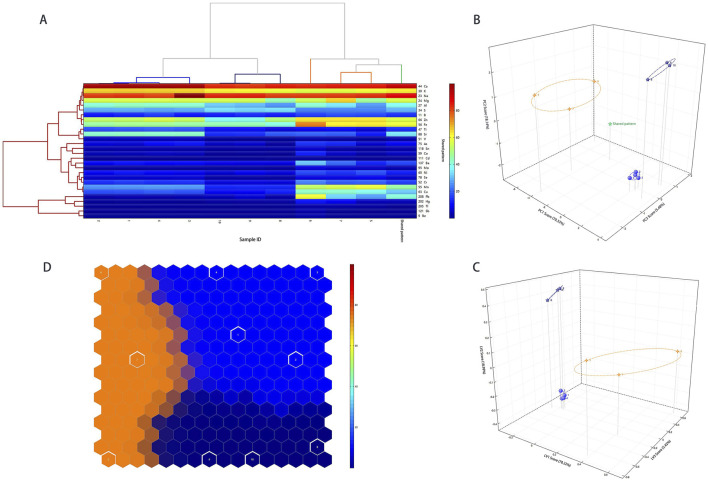
The results of the validation model. Dendrograms of the HCA **(A)**; score plots of the PCA model **(B)**; score plots of the PLS-DA model **(C)**; self-organizing mapping artificial neural network analysis **(D)**.

To verify the experimental model, the aforementioned 25 samples were used as the training set, and 10 samples collected were used as the test set. The results of the validation model of HCA, PCA, PLS-DA, and SOM (Figure 3) showed that the three Calculus bovis samples were clustered into three categories, consistent with the categories of Calculus bovis. This further verified that metal fingerprinting plays a significant role in distinguishing the three types of Calculus bovis, which obtained 100% classification for all samples used in this study.

### 3.2 Strategy of the risk–benefit assessment based on the three species of the Calculus bovis

#### 3.2.1 Results of the non-carcinogenic risk assessment

The HI results of heavy metals in the three species of Calculus bovis are shown in Tables 2–4. In the assessment results for medicinal and edible purposes, for Bovis calculus artifactus, in both male and female groups, the HI mean values for different metal elements were in the order Be < Cu < Ba < V < Al < Cd < Mn < Ni < Pb < Hg < As, the HI minimal values for different metal elements were in the order Be < Cu < Cd < V < Ba < Al < Mn < Pb < Ni < Hg < As, and the HI maximal values for different metal elements were in the order Cu < Ba < Be < V < Al < Mn < Cd < Ni < Hg < Pb < As; for Calculus bovis, the HI mean values for different metal elements in both male and female groups were in the order V < Be < Al < Ba < Cd < Cu < Ni < Hg < Mn < As < Pb; the minimum HI values for different metal elements followed the order Be < Ba < Al < V < Cu < Cd < Ni < Hg < Pb < Mn < As; the maximum HI values for different metal elements were in the order V < Al < Be < Cd < Ba < Cu < Ni < Hg < Mn < As < Pb; for Bovis calculus sativus, in both male and female populations, the HI mean values for different metal elements followed the order Ba < Be < Cu < Cd < V < Al < Hg < Ni < Al < Mn < As; the minimum HI for different metallic elements were in the order Be < Ba < Cu < Cd < V < Ni < Pb < Hg < Al < Mn < As; and the maximum HI for different metallic elements were in the order Ba < Cu < Be < Cd < V < Hg < Pb < Ni < Al < Mn < As. The HI results showed that HI values of Pb in Calculus bovis were higher than 1 for both male and female individuals.

**TABLE 2:** The mean of HI values of metal elements in Calculus bovis of different species used for medicinal and food purposes.

Element	Bovis calculus artifactus				Bovis calculus				Bovis calculus sativus			
	Food		TCM		Food		TCM		Food		TCM	
	Male	Female	Male	Female	Male	Female	Male	Female	Male	Female	Male	Female
Cu	2.12E-05	2.50E-05	1.47E-05	1.73E-05	6.96E-04	8.20E-04	4.82E-04	5.68E-04	6.39E-05	7.54E-05	4.42E-05	5.22E-05
Mn	1.34E-04	1.58E-04	9.26E-05	1.09E-04	1.60E-02	1.89E-02	1.11E-02	1.31E-02	6.27E-04	7.40E-04	4.34E-04	5.12E-04
V	4.66E-05	5.50E-05	3.23E-05	3.81E-05	1.06E-04	1.25E-04	7.32E-05	8.64E-05	3.14E-04	3.71E-04	2.18E-04	2.57E-04
Ni	4.20E-04	4.95E-04	2.91E-04	3.43E-04	1.20E-03	1.42E-03	8.32E-04	9.82E-04	4.41E-04	5.20E-04	3.05E-04	3.60E-04
Ba	3.31E-05	3.90E-05	2.29E-05	2.70E-05	2.65E-04	3.13E-04	1.83E-04	2.16E-04	4.64E-05	5.47E-05	3.21E-05	3.79E-05
Be	1.79E-05	2.11E-05	1.24E-05	1.46E-05	1.66E-04	1.96E-04	1.15E-04	1.36E-04	5.63E-05	6.64E-05	3.90E-05	4.60E-05
Al	8.96E-05	1.06E-04	6.21E-05	7.32E-05	1.84E-04	2.17E-04	1.27E-04	1.50E-04	5.25E-04	6.19E-04	3.64E-04	4.29E-04
As	9.93E-03	1.17E-02	6.87E-03	8.11E-03	3.38E-02	3.99E-02	2.34E-02	2.76E-02	1.65E-02	1.95E-02	1.14E-02	1.35E-02
Cd	1.07E-04	1.27E-04	7.44E-05	8.78E-05	4.71E-04	5.55E-04	3.26E-04	3.84E-04	2.35E-04	2.78E-04	1.63E-04	1.92E-04
Hg	1.05E-03	1.24E-03	7.26E-04	8.57E-04	4.59E-03	5.41E-03	3.18E-03	3.75E-03	4.30E-04	5.07E-04	2.98E-04	3.51E-04
Pb	5.89E-04	6.95E-04	4.08E-04	4.81E-04	3.21E-01	3.78E-01	2.22E-01	2.62E-01	4.25E-04	5.02E-04	2.95E-04	3.47E-04
K	1.41E-01	9.74E-02	9.75E-02	9.74E-02	1.09E-01	6.42E-02	7.58E-02	6.42E-02	2.82E-01	1.66E-01	1.95E-01	1.66E-01
Ca	2.64E+00	1.83E+00	1.83E+00	1.83E+00	2.61E+00	1.53E+00	1.81E+00	1.53E+00	9.88E+00	5.79E+00	6.84E+00	5.79E+00
Na	2.84E+00	1.97E+00	1.96E+00	1.97E+00	2.13E+00	1.25E+00	1.48E+00	1.25E+00	2.18E+00	1.28E+00	1.51E+00	1.28E+00
Mg	2.80E-01	1.92E-01	1.94E-01	1.92E-01	1.18E+00	6.87E-01	8.17E-01	6.87E-01	5.83E-01	3.39E-01	4.04E-01	3.39E-01
Fe	7.98E-01	5.49E-01	5.52E-01	5.49E-01	9.48E+00	5.53E+00	6.56E+00	5.53E+00	1.54E+00	8.97E-01	1.06E+00	8.97E-01
Zn	1.32E+01	6.38E-02	9.15E+00	9.55E+00	9.90E+00	1.78E+00	6.85E+00	6.07E+00	3.36E+00	1.63E-01	2.32E+00	2.06E+00
Cr	2.78E-02	9.55E+00	1.93E-02	1.79E-02	1.13E-01	6.07E+00	7.85E-02	6.17E-02	4.48E-02	2.06E+00	3.10E-02	2.44E-02
Se	2.19E-04	1.79E-02	1.52E-04	1.50E-04	1.13E-03	6.17E-02	7.85E-04	6.60E-04	8.39E-04	2.44E-02	5.81E-04	4.88E-04
Mo	4.52E-05	1.50E-04	3.13E-05	3.12E-05	3.80E-04	6.60E-04	2.63E-04	2.23E-04	6.78E-05	4.88E-04	4.70E-05	3.97E-05

**TABLE 3:** The maximum of HI values of metal elements in Calculus bovis of different species used for medicinal and food purposes.

Element	Bovis calculus artifactus				Bovis calculus				Bovis calculus sativus			
	Food		TCM		Food		TCM		Food		TCM	
	Male	Female	Male	Female	Male	Female	Male	Female	Male	Female	Male	Female
Cu	2.12E-05	2.50E-05	1.47E-05	1.73E-05	6.96E-04	8.20E-04	4.82E-04	5.68E-04	6.39E-05	7.54E-05	4.42E-05	5.22E-05
Mn	1.34E-04	1.58E-04	9.26E-05	1.09E-04	1.60E-02	1.89E-02	1.11E-02	1.31E-02	6.27E-04	7.40E-04	4.34E-04	5.12E-04
V	4.66E-05	5.50E-05	3.23E-05	3.81E-05	1.06E-04	1.25E-04	7.32E-05	8.64E-05	3.14E-04	3.71E-04	2.18E-04	2.57E-04
Ni	4.20E-04	4.95E-04	2.91E-04	3.43E-04	1.20E-03	1.42E-03	8.32E-04	9.82E-04	4.41E-04	5.20E-04	3.05E-04	3.60E-04
Ba	3.31E-05	3.90E-05	2.29E-05	2.70E-05	2.65E-04	3.13E-04	1.83E-04	2.16E-04	4.64E-05	5.47E-05	3.21E-05	3.79E-05
Be	1.79E-05	2.11E-05	1.24E-05	1.46E-05	1.66E-04	1.96E-04	1.15E-04	1.36E-04	5.63E-05	6.64E-05	3.90E-05	4.60E-05
Al	8.96E-05	1.06E-04	6.21E-05	7.32E-05	1.84E-04	2.17E-04	1.27E-04	1.50E-04	5.25E-04	6.19E-04	3.64E-04	4.29E-04
As	9.93E-03	1.17E-02	6.87E-03	8.11E-03	3.38E-02	3.99E-02	2.34E-02	2.76E-02	1.65E-02	1.95E-02	1.14E-02	1.35E-02
Cd	1.07E-04	1.27E-04	7.44E-05	8.78E-05	4.71E-04	5.55E-04	3.26E-04	3.84E-04	2.35E-04	2.78E-04	1.63E-04	1.92E-04
Hg	1.05E-03	1.24E-03	7.26E-04	8.57E-04	4.59E-03	5.41E-03	3.18E-03	3.75E-03	4.30E-04	5.07E-04	2.98E-04	3.51E-04
Pb	5.89E-04	6.95E-04	4.08E-04	4.81E-04	3.21E-01	3.78E-01	2.22E-01	2.62E-01	4.25E-04	5.02E-04	2.95E-04	3.47E-04
K	1.41E-01	9.74E-02	9.75E-02	9.74E-02	1.09E-01	6.42E-02	7.58E-02	6.42E-02	2.82E-01	1.66E-01	1.95E-01	1.66E-01
Ca	2.64E+00	1.83E+00	1.83E+00	1.83E+00	2.61E+00	1.53E+00	1.81E+00	1.53E+00	9.88E+00	5.79E+00	6.84E+00	5.79E+00
Na	2.84E+00	1.97E+00	1.96E+00	1.97E+00	2.13E+00	1.25E+00	1.48E+00	1.25E+00	2.18E+00	1.28E+00	1.51E+00	1.28E+00
Mg	2.80E-01	1.92E-01	1.94E-01	1.92E-01	1.18E+00	6.87E-01	8.17E-01	6.87E-01	5.83E-01	3.39E-01	4.04E-01	3.39E-01
Fe	7.98E-01	5.49E-01	5.52E-01	5.49E-01	9.48E+00	5.53E+00	6.56E+00	5.53E+00	1.54E+00	8.97E-01	1.06E+00	8.97E-01
Zn	1.32E+01	6.38E-02	9.15E+00	9.55E+00	9.90E+00	1.78E+00	6.85E+00	6.07E+00	3.36E+00	1.63E-01	2.32E+00	2.06E+00
Cr	2.78E-02	9.55E+00	1.93E-02	1.79E-02	1.13E-01	6.07E+00	7.85E-02	6.17E-02	4.48E-02	2.06E+00	3.10E-02	2.44E-02
Se	2.19E-04	1.79E-02	1.52E-04	1.50E-04	1.13E-03	6.17E-02	7.85E-04	6.60E-04	8.39E-04	2.44E-02	5.81E-04	4.88E-04
Mo	4.52E-05	1.50E-04	3.13E-05	3.12E-05	3.80E-04	6.60E-04	2.63E-04	2.23E-04	6.78E-05	4.88E-04	4.70E-05	3.97E-05

**TABLE 4:** The minimum of HI values of metal elements in Calculus bovis of different species used for medicinal and food purposes.

Element	Bovis calculus artifactus				Bovis calculus				Bovis calculus sativus			
	Food		TCM		Food		TCM		Food		TCM	
	Male	Female	Male	Female	Male	Female	Male	Female	Male	Female	Male	Female
Cu	1.72E-05	2.03E-05	1.19E-05	1.40E-05	2.95E-04	3.49E-04	2.05E-04	2.41E-04	6.02E-05	7.10E-05	4.17E-05	4.92E-05
Mn	1.13E-04	1.33E-04	7.82E-05	9.22E-05	9.42E-03	1.11E-02	6.52E-03	7.69E-03	5.90E-04	6.96E-04	4.09E-04	4.82E-04
V	2.90E-05	3.42E-05	2.01E-05	2.37E-05	6.71E-05	7.91E-05	4.64E-05	5.48E-05	2.91E-04	3.43E-04	2.01E-04	2.37E-04
Ni	2.54E-04	3.00E-04	1.76E-04	2.08E-04	6.29E-04	7.42E-04	4.35E-04	5.13E-04	3.49E-04	4.12E-04	2.42E-04	2.85E-04
Ba	2.99E-05	3.53E-05	2.07E-05	2.44E-05	3.75E-05	4.42E-05	2.60E-05	3.06E-05	3.94E-05	4.65E-05	2.73E-05	3.22E-05
Be	0.00E+00	0.00E+00	0.00E+00	0.00E+00	7.68E-06	9.06E-06	5.31E-06	6.27E-06	3.07E-05	3.62E-05	2.13E-05	2.51E-05
Al	3.56E-05	4.20E-05	2.47E-05	2.91E-05	5.74E-05	6.77E-05	3.97E-05	4.69E-05	5.00E-04	5.90E-04	3.46E-04	4.08E-04
As	3.14E-03	3.70E-03	2.17E-03	2.56E-03	1.28E-02	1.51E-02	8.83E-03	1.04E-02	1.13E-02	1.33E-02	7.82E-03	9.22E-03
Cd	2.05E-05	2.41E-05	1.42E-05	1.67E-05	3.48E-04	4.10E-04	2.41E-04	2.84E-04	2.15E-04	2.54E-04	1.49E-04	1.76E-04
Hg	9.31E-04	1.10E-03	6.45E-04	7.61E-04	9.77E-04	1.15E-03	6.77E-04	7.98E-04	3.84E-04	4.53E-04	2.66E-04	3.13E-04
Pb	1.81E-04	2.14E-04	1.26E-04	1.48E-04	6.94E-03	8.19E-03	4.80E-03	5.67E-03	3.51E-04	4.14E-04	2.43E-04	2.87E-04
K	1.13E-01	7.79E-02	7.79E-02	7.79E-02	8.89E-02	5.21E-02	6.15E-02	5.21E-02	2.61E-01	1.53E-01	1.81E-01	1.53E-01
Ca	1.89E+00	1.31E+00	1.31E+00	1.31E+00	2.32E+00	1.36E+00	1.61E+00	1.36E+00	9.47E+00	5.55E+00	6.56E+00	5.55E+00
Na	2.04E+00	1.41E+00	1.41E+00	1.41E+00	1.92E+00	1.13E+00	1.33E+00	1.13E+00	1.95E+00	1.14E+00	1.35E+00	1.14E+00
Mg	1.03E-01	7.08E-02	7.15E-02	7.08E-02	2.03E-01	1.18E-01	1.41E-01	1.18E-01	5.51E-01	3.20E-01	3.81E-01	3.20E-01
Fe	3.97E-01	2.73E-01	2.75E-01	2.73E-01	7.32E-01	4.27E-01	5.06E-01	4.27E-01	1.40E+00	8.20E-01	9.72E-01	8.20E-01
Zn	4.93E-01	5.18E-02	3.42E-01	3.57E-01	7.39E+00	7.54E-01	5.12E+00	4.53E+00	3.05E+00	1.54E-01	2.11E+00	1.87E+00
Cr	1.30E-02	3.57E-01	8.97E-03	8.33E-03	7.40E-02	4.53E+00	5.12E-02	4.03E-02	3.75E-02	1.87E+00	2.60E-02	2.04E-02
Se	1.97E-04	8.33E-03	1.36E-04	1.35E-04	9.63E-04	4.03E-02	6.67E-04	5.61E-04	7.41E-04	2.04E-02	5.13E-04	4.31E-04
Mo	3.05E-05	1.35E-04	2.11E-05	2.11E-05	2.18E-04	5.61E-04	1.51E-04	1.28E-04	5.83E-05	4.31E-04	4.04E-05	3.41E-05

#### 3.2.2 Results of the carcinogenic risk assessment

Studies have shown that the toxicity of elements is closely related to their speciation. To protect most consumers, this study assumed that all As speciations were the most toxic inorganic arsenic. The corresponding carcinogenic risk of Calculus bovis was calculated according to Equation 3. The results showed that the CRs of Pb and As in the three Calculus bovis items were less than 1 × 10^−4^, indicating that the risks were acceptable.

#### 3.2.3 Results of the risk–benefit assessment

The study results for the nutrient elements were subjected to the principle of elemental risk–benefit assessment. In the risk–benefit assessment results of Bovis calculus artifactus for medicinal and edible purposes, in both male and female groups, the mean, minimum, and maximum values of HI were greater than 1 for Ca, Na, and Zn, and the mean, minimum, and maximum values of BI were less than 1 for K, Mg, Cr, Se, and Mo, indicating that the health risks of Ca, Na, and Zn were higher than the health benefits in the Bovis calculus artifactus; the health risks of Ca, Na, and Zn were higher, and the health benefits of K, Mg, Cr, Se, and Mo were lower. This situation deserves attention. Furthermore, the risk–benefit level of the Fe element was maintained in balance; for Calculus bovis, in the male group, the HI mean, minimum, and maximum values of Ca, Na, Cu, and Zn were all greater than 1, the mean and maximum values were greater than 1 for elemental Ca, Na, and Zn and greater than 1 for elemental Fe and Cu in the female group; in both male and female groups, the BI mean, minimum, and maximum values for K, Cr, Se, and Mo were less than 1. The BI mean and minimum values for Mg were less than 1, indicating that, in Calculus bovis, the health risks of Ca, Na, Cu, Zn, and Fe were higher and the health benefits of K, Cr, Se, Mo, and Mg were lower; the results deserve attention. For Bovis calculus sativus, the HI mean, minimum, and maximum values for both male and female populations for Ca, Na, and Zn were greater than 1. Those of Fe were greater than 1, and those of K, Mg, Se, and Mo were less than 1. The mean, minimum, and maximum values of BI for K, Mg, Cr, Se, and Mo were all less than 1, indicating that the health risks of Ca, Na, Zn, and Fe were higher, the health benefits of K, Mg, Cr, Se, and Mo were lower, and the risk–benefit level of Cu was maintained at an equilibrium state in Bovis calculus sativus, which needs attention.

## 4 Discussion

The knowledge and understanding of medicines and food supplements are usually based on their color, shape, taste, and content of active ingredients. However, the importance of chemical elements has still been ignored. The efficacy of medicines and food supplements is closely related to the presence of inorganic elements. Inorganic elements are divided into macronutrients, trace elements, and harmful elements. Macronutrients mainly include K, Ca, Na, and Mg. K can be adjusted by regulating osmolality to dynamically regulate the body’s acid–base balance (Ankit et al., 2022). Ca is the most abundant mineral element in the human body and is important in the development of the human skeleton and the maintenance of normal physiological function of the heart. Calcium is also an important messenger in development of many diseases (Khan et al., 2023). Na promotes the metabolism of the human body. It maintains the stability of intracellular water to maintain the health and balance of the human body (Neugebauer et al., 2022). Mg, an electrolyte in the body, is important in forming bones and teeth and maintaining normal neuromuscular functions (Arancibia-Hernández et al., 2023). Trace elements mainly include Fe, Cu, Zn, Mn, Cr, and 16 other elements, with different elements having different functions; for example, Fe is involved in the synthesis of hemoglobin and oxygen transportation; a lack of iron may result in dizziness, fatigue, and other symptoms of maladaptation (Fitsanakis et al., 2010). Cu plays a role in regulating normal functions of the heart rate, blood pressure, bones, connective tissues, and viscera; copper deficiency may lead to skin pallor, premature gray hair, bone development disorders, and other symptoms (Zhong et al., 2022). In addition, there are harmful elements in TCMs and food supplements, mainly lead, cadmium, arsenic, mercury, and eight other elements, for which a reasonable risk assessment should be carried out to clarify the extent of the harmful elements, providing a reference for the scientific cultivation and application of CHMs.

Chemometrics, an interdisciplinary field combining statistics and computer technology, has played a crucial role in this study. Through the preprocessing of elemental data from different types of Calculus bovis species and the application of pattern recognition techniques, we successfully achieved the classification and identification of various types of Calculus bovis. This approach holds significant potential for future applications such as food authenticity verification and traceability of origin. Compared to other certification methods, chemometrics is more effective in handling complex nonlinear relationships (Liu et al., 2019). Furthermore, the application of chemometrics has a significant impact on regulatory standards. By using chemometric methods, the composition of chemical substances can be detected and quantified more accurately, providing technical support for the development and enforcement of stricter regulatory standards.

**FIGURE 4:**
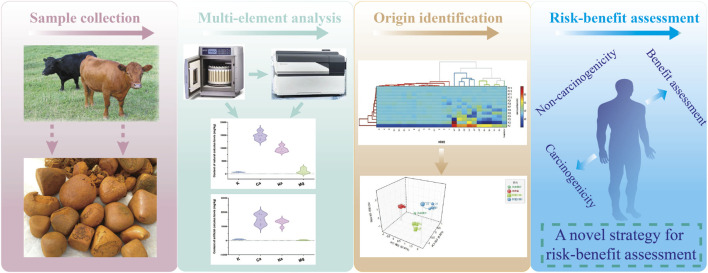
Schematic diagram of Calculus bovis in this study.

Based on the traditional risk assessment, this study is the first to comprehensively adopt the heavy metal and nutrient element multi-element risk–benefit evaluation model relevant to using TCMs to evaluate multi-element risk–benefit in three Calculus bovis products. The multi-element risk–benefit evaluation method for TCMs established in this study consists of four steps: the identification of hazards, the characterization of hazards, the assessment of exposures, and the description of the risk–benefit. To ensure the rationality and accuracy of the evaluation, we included gender as one of the conditions for evaluation. In addition, we used the minimum, average, and maximum values of HI and BI in evaluating the three Calculus bovis products. This method broadens the scope of risk assessment, covers both hazardous and nutrient elements, and provides a comprehensive evaluation of the health risks and benefits that may affect the human body. This innovative multi-element method meets the requirements of most elements and is consistent with the risk–benefit evaluation of TCMs (Figure 4).

This study has certain limitations. For instance, due to the difficulty in obtaining Calculus bovis samples, the sample size used in this study was relatively small. In future research, we aim to obtain a larger number of samples to support the results with more data. Second, regarding the multiple elements in Calculus bovis, we did not consider the elemental speciation as different speciation forms often exhibit significantly different toxicities. In addition, we conducted the risk–benefit assessment based on the total elemental contents of heavy metals without considering the actual amount absorbed by the human bodies, i.e., bioavailability. Actually, the bioavailability of elements may not be 100%, which may lead to an overestimation of the risks or benefits of samples. Therefore, in future studies, we will incorporate the various speciations and bioavailability of elements into the actual risk–benefit assessment to obtain more accurate evaluation results.

## 5 Conclusion

We identified three Calculus bovis products using ICP-MS technology combined with chemometric analysis. The results indicated that this approach distinguishes the three Calculus bovis products according to their content and distribution of inorganic elements; it establishes their fingerprints of the characteristic elements. Furthermore, a multi-element risk–benefit evaluation of heavy metals and nutrients was performed to comprehensively evaluate the risk–benefit profile of multi-elements present in three Calculus bovis products. This study provides a new research model for the identification of the herbs and substitutes of Calculus bovis, provides new ideas for the quality control of Calculus bovis and sustainable utilization of resources, and has a practical significance and reference value for the scientific and rational application of Calculus bovis.

## Data Availability

The original contributions presented in the study are included in the article/Supplementary Material; further inquiries can be directed to the corresponding authors.
