# Supplementary material for: Metal element fingerprints combined with chemometrics deciphering the discrimination of different Calculus bovis and a novel risk–benefit assessment
Source: Front Chem. 2026 Feb 10;14:1752261. doi: 10.3389/fchem.2026.1752261 (PMC12929478; doi:10.3389/fchem.2026.1752261)
Supplement: Supplementary file 1 [file Table1.docx]

**Supplementary Material**

**Table S1**

**LODs ~~and~~ LOQs and recoveries of elements（μg·kg^-1^，*n*=3）**

| Elements | LOD | LOQ | Recoveries(%) |
| --- | --- | --- | --- |
| Al | 0.009 | 0.031 | 85.8±1.2 |
| As | 0.005 | 0.017 | 92.3±2.3 |
| Au | 0.005 | 0.017 | 87.3±2.8 |
| B | 0.023 | 0.070 | 105.3±3.2 |
| Ba | 0.030 | 0.090 | 92.8±2.8 |
| Be | 0.003 | 0.009 | 94.3±1.2 |
| Ca | 0.033 | 0.098 | 102.3±0.2 |
| Cd | 0.002 | 0.007 | 99.2±3.9 |
| Co | 0.002 | 0.006 | 112.3±0.8 |
| Cr | 0.009 | 0.027 | 87.2±2.2 |
| Cu | 0.004 | 0.013 | 102.3±2.3 |
| Fe | 0.009 | 0.031 | 112.3±0.2 |
| Hg | 0.007 | 0.024 | 102.3±0.8 |
| K | 0.009 | 0.028 | 112.2±1.2 |
| Mg | 0.025 | 0.082 | 109.9±0.9 |
| Mn | 0.005 | 0.017 | 87.2±2.2 |
| Mo | 0.003 | 0.009 | 89.7±3.2 |
| Na | 0.042 | 0.139 | 104.2±0.8 |
| Ni | 0.006 | 0.019 | 111.3±1.1 |
| Pb | 0.002 | 0.007 | 104.7±2.3 |
| Sb | 0.016 | 0.055 | 88.3±2.7 |
| Se | 0.015 | 0.047 | 92.7±3.3 |
| Sr | 0.002 | 0.007 | 97.3±0.9 |
| Ti | 0.002 | 0.008 | 102.6±1.6 |
| Tl | 0.002 | 0.007 | 85.9±2.2 |
| V | 0.002 | 0.007 | 93.2±3.9 |
| Zn | 0.023 | 0.076 | 95.3±2.9 |
